# *Cudrania tricuspidata* Combined with *Lacticaseibacillus rhamnosus* Modulate Gut Microbiota and Alleviate Obesity-Associated Metabolic Parameters in Obese Mice

**DOI:** 10.3390/microorganisms9091908

**Published:** 2021-09-08

**Authors:** Ju Kyoung Oh, Robie Vasquez, In-Chan Hwang, Ye Na Oh, Sang Hoon Kim, Shin Ho Kang, Jae Yeon Joung, Nam Su Oh, Sejeong Kim, Yohan Yoon, Dae-Kyung Kang

**Affiliations:** 1 Department of Animal Resources Science, Dankook University, Cheonan 31116, Korea; ju.kyoung.oh@ki.se (J.K.O.); robie.vasquez1@gmail.com (R.V.); hec777@naver.com (I.-C.H.); oyena0609@naver.com (Y.N.O.); giooman@naver.com (S.H.K.); 2 R&D Center, Seoul Dairy Cooperative, Ansan 15407, Korea; shkang@seoulmilk.co.kr (S.H.K.); fyd0123@seoulmilk.co.kr (J.Y.J.); 3 Department of Food and Biotechnology, Korea University Sejong Campus, Sejong 30019, Korea; klanvin@korea.ac.kr; 4 Department of Food and Nutrition, Sookmyung Women’s University, Seoul 04310, Korea; skim@sookmyung.ac.kr (S.K.); yyoon@sookmyung.ac.kr (Y.Y.)

**Keywords:** obesity, *Lacticaseibacillus rhamnosus* 4B15, *Cudrania tricuspidata*, gut microbiota, metabolic disorder, probiotic, prebiotic, synbiotic

## Abstract

The aim of the presented study was to investigate the synbiotic effects of *L. rhamnosus* 4B15 and *C. tricuspidata* extract administration on the gut microbiota and obesity-associated metabolic parameters in diet-induced obese mice. Thirty-one 6-week-old male C57BL/N6 mice were divided into five diet groups: normal diet (ND, *n* = 7) group; high-fat diet (HFD, *n* = 6) group; probiotic (PRO, *n* = 5) group; prebiotic (PRE, *n* = 7) group; and synbiotic (SYN, *n* = 6) group. After 10 weeks, the percent of fat mass, serum triglyceride, and ALT levels were significantly reduced in SYN-fed obese mice, compared with other treatments. SYN treatment also modulated the abundance of *Desulfovibrio*, *Dorea*, *Adlercreutzia*, *Allobaculum*, *Coprococcus*, unclassified Clostridiaceae, *Lactobacillus*, *Helicobacter*, *Flexispira*, *Odoribacter*, *Ruminococcus*, unclassified Erysipelotrichaceae, and unclassified Desulfovibrionaceae. These taxa showed a strong correlation with obesity-associated indices. Lastly, the SYN-supplemented diet upregulated metabolic pathways known to improve metabolic health. Further investigations are needed to understand the mechanisms driving the synbiotic effect of *C. tricuspidata* and *L. rhamnosus* 4B15.

## 1. Introduction

In recent years, the strong relationship between the gut microbiota and the host’s physiology and health have been fairly established [1,2,3,4]. Several studies have demonstrated the role of the gut microbiota in the host’s metabolism, such that its modulation would affect its host beneficially or otherwise [2,3,4,5]. Aside from environment and genetic factors, diet plays a critical role in shaping the host’s gut microbiota [1,6]; thus, researchers have focused on modulating the gut microbiota through diet. The potential of probiotics and prebiotics in influencing the gut microbiota has been the subject of recent studies [7,8]. Probiotics are live, beneficial microorganisms that can improve microbial balance in the intestinal tract [9,10]. Probiotics have shown great potential for alleviating obesity, metabolic diseases, preventing food allergy and neurodegenerative disorders, as well as elimination of the pathogenic *Helicobacter* [11,12,13,14]. Hence, there is now a great interest in food containing probiotics, as well as fermented food [15,16]. Prebiotics, on the other hand, are a non-digestible substrate which may include oligosaccharides and dietary fibres [7,8,14,17]. The supplementation of prebiotics can beneficially affect host metabolism through gut microbiota modulation [14,18]. Multiple studies have shown the promising effect of both probiotics and prebiotics, and its combination (synbiotics) in the promotion of gut health, as well as the alleviation of metabolic disorders [7,14,19,20,21]. Synbiotic application in several animal models demonstrated promising results in the amelioration of intestinal inflammation and obesity, control of cholesterol, prevention of diarrhoea, and improvement of host metabolism [22,23,24,25,26].

*Lacticaseibacillus rhamnosus* 4B15, a lactobacillus strain recently isolated from infant faeces, has probiotic and anti-inflammatory potential [27]. On the other hand, *Cudrania tricuspidata*, a tree endemic to Korea and other parts of East Asia, is an important and widely used herbal medicine in Korea [28]. Investigations have shown that *C. tricuspidata* extracts can alleviate excess adiposity and serum triglyceride levels, and ameliorate insulin resistance and hyperglycaemia in murine models [28,29]. Another study revealed that *C. tricuspidata* extracts exhibit anti-obesity and antidiabetic potential by inhibiting protein-tyrosine phosphatase 1B (PTP1B), an important metabolic syndrome marker [30]. The synbiotic potential of *L. rhamnosus* 4B15 and *C. tricuspidata* in modulation of gut microbiota and the alleviation of metabolic disorders has not been studied. Therefore, the goal of this study was to investigate the effects of *L. rhamnosus* 4B15 and *C. tricuspidata* extract administration on the gut microbiota and obesity-associated metabolic parameters in diet-induced obese mice.

## 2. Material and Methods

### 2.1. Animals and Experimental Design

Thirty-one 6-week-old male C57BL/N6 mice were divided into five diet groups (5–7 mice per group, 2–3 mice per cage): normal diet (ND) group (*n* = 7); high-fat diet (HFD) group (*n* = 6); probiotic HFD (PRO) group (*n* = 5); prebiotic HFD (PRE) group (*n* = 7); and synbiotic HFD (SYN) group (*n* = 6). The ND group was provided a diet containing 10% fat, while the HFD group was provided an HFD (45% fat) (Table S1). The PRO, PRE, and SYN groups were also provided an HFD (45% fat) supplemented with either the probiotic *L. rhamnosus* 4B15, the prebiotic *C. tricuspidata*, or a combination of the prebiotic and probiotic (Figure S1). *C. tricuspidata* leaves were obtained from a local market in Sunchang, Jeollakbu-do, the Republic of Korea, while *L. rhamnosus* 4B15 was obtained from the Korean Culture Centre of Microorganisms (KCCM11983P). *C. tricuspidata* leaf extract and *L. rhamnosus* 4B15 were prepared according to previous methods [31,32]. The treatments were administered through oral gavage daily (200 µL/day). The concentrations of *L. rhamnosus* 4B15 and *C. tricuspidata* extract were 1.0 × 10^9^ CFU/g and 1500 mg/mL, respectively (Figure S1). Mouse cages were equipped with a one-sided self-feeder and a nipple water-feeder for *ad libitum* access to feed and water throughout the experiment. The body weights of the mice were measured weekly. After 10 weeks of feeding, the mice were sacrificed; intestine, fat, and serum samples were collected. All animal protocols were approved by the Sookmyung Women’s University Animal Care Committee (SMWU-IACUC-1703-001-01; Seoul, South Korea; 26 December 2017).

### 2.2. Total Fat Mass and Serum Biochemical Analysis

After sacrifice, epididymal and inguinal fat were collected and weighed. The percent of fat mass was calculated using the formula: total fat mass/body weight × 100. The blood samples were placed in serum-separating tubes (Microtainer, Becton, Dickinson and Company, NJ, USA), followed by centrifugation at 2339× *g* for 10 min at 4 °C. The supernatants were then transferred to sterile micro-centrifuge tubes, immediately frozen and stored at −70 °C until further analyses. A chemistry analyser (Mindray, Shenzhen, China) was used to measure the serum triglyceride (TG), total cholesterol (T-Chol), and alanine aminotransferase activity (ALT) levels.

### 2.3. DNA Extraction and Sequencing

The mouse intestines were immediately transported to the laboratory at 4 °C, where they were stored in a deep freezer at −70 °C until further analysis. Genomic DNA was extracted using a QIAamp Power Fecal DNA Isolation Kit (Qiagen, Hilden, Germany) following the manufacturer’s instructions. The concentration and purity of the extracted genomic DNA were confirmed by agarose gel electrophoresis and UV-visible spectrophotometry (Mecasys Co., Ltd., Daejeon, Republic of Korea). PCR amplification of 16S rRNA was performed using primers for the V3–V4 hypervariable region. The PCR conditions were as follows: initial denaturation at 94 °C for 5 min, followed by 30 cycles of denaturation at 94 °C for 30 s, annealing at 55 °C for 45 s, and final extension at 72 °C for 1 min 30 s. The amplicons were purified using a NucleoSpin Clean-up Kit (Macherey-Nagel, Dueren, Germany) following the manufacturer’s instructions, and then used to construct sequencing libraries with an Illumina TruSeq DNA Sample Preparation Kit (Illumina, San Diego, CA, USA). For each sample, barcoded V3–V4 PCR amplicons were sequenced using the Illumina Miseq platform (MiSeq Reagent Kit v2, Illumina, CA, USA). Finally, amplification and sequencing of the V3–V4 region of the 16S rRNA gene was performed by ChunLab, Inc. (Seoul, Republic of Korea).

### 2.4. Microbial Community Analysis

Raw sequence data generated by the Illumina MiSeq platform were processed using the Quantitative Insights into Microbial Ecology (QIIME, version 1.9.0) pipeline [33]. Briefly, sequences that were less than 200 bp or greater than 600 bp in length, of low quality, or that had incorrect primer sequences and/or contained more than one ambiguous base were filtered out using the split_libraries.py script. Chimeric sequences were identified using the identify_chimeric_seqs.py and filter_fasta.py scripts, and removed from the sequence data. Operational taxonomic units were identified using the pick_open_reference.py script and the most recent Greengenes database (13_8); a 97% identity threshold was used [34].

Microbial composition data from mouse intestine samples were generated using the summarize_taxa_through_plots.py script. For alpha-diversity indices, the alpha_diversity.py script was employed to generate ACE, Chao1, Shannon–Weaver, and Simpson values. To illustrate the differences among the groups, a principal component analysis (PCA) based on the Bray–Curtis matrix was performed using the R software (v.4.0.2; R Development Core Team, Vienna, Austria). To compare the relative abundance of groups, boxplots were generated using the GraphPad Prism software (ver. 8.4.2; GraphPad Software Inc., San Diego, CA, USA). To identify and visualize the core microbiota for all groups, Venn diagrams were produced using an online tool (http://bioinformatics.psb.ugent.be/webtools/Venn/; accessed on 6 May 2021).

### 2.5. Bioinformatic Analysis

To determine differential taxa, a linear discriminant analysis effect size (LEfSe) [35] analysis was performed using an online tool (https://huttenhower.sph.harvard.edu/galaxy/; accessed on 9 May 2021). Briefly, the LEfSe parameters were as follows: (1) the alpha value for the Kruskal–Wallis test of differences among classes <0.05; (2) the alpha value for the pairwise Wilcoxon test of differences among subclasses <than 0.05; (3) the threshold for the logarithmic linear discriminant analysis (LDA) score of 2.0; and (4) the all-against-all strategy for multi-class analysis. To predict functional pathways in the microbiome, phylogenetic investigation of communities by reconstruction of unobserved states (PICRUSt) [36] was performed based on the Kyoto Encyclopaedia of Genes and Genomes (KEGG) (level 3) using an online tool (https://huttenhower.sph.harvard.edu/galaxy/; accessed on 4 May 2021).

### 2.6. Statistical Analysis

Statistical analyses were performed using GraphPad Prism (ver. 8.4.2). The normality of the data distribution was analysed using the Shapiro–Wilk test. A one-way ANOVA with a post-hoc Tukey HSD or Kruskal–Wallis test was used to analyse differences in body weight, metabolic parameters, relative abundance and alpha diversity. To determine whether the gut microbial distribution differed among groups, permutational multivariate analysis of variance (PERMANOVA) was performed using R (v.4.0.2). Significantly different KEGG pathways were identified using STAMP [37]. The correlation between obesity associated metabolic factors and gut microbiota was analysed using Spearman’s rank correlation in R (v.4.0.2). In all statistical calculations, differences were considered significant at *p* < 0.05, *p* < 0.01, and *p* < 0.001.

## 3. Results

### 3.1. Effect of Administration L. rhamnosus 4B15 and C. tricuspidata on Body Weight and Metabolic Parameters in Obese Mice

**Figure 1:**
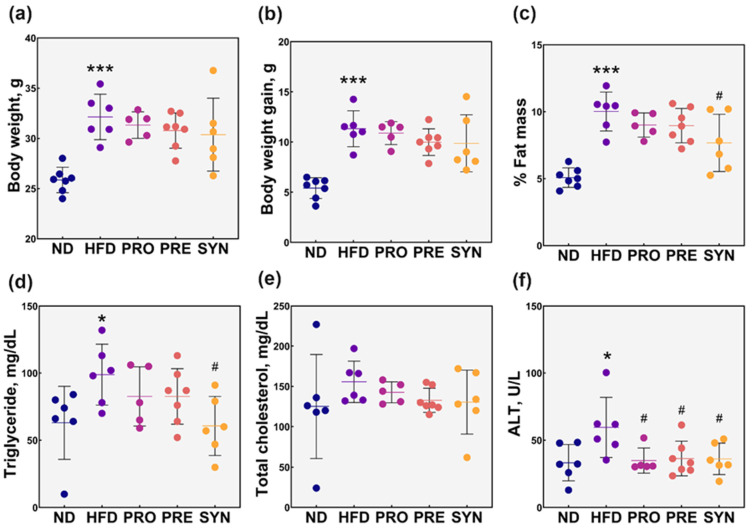
Effect of administration of *L. rhamnosus* 4B15 and *C. tricuspidata* on body weight and metabolic parameters. Comparison of the effect of each treatment on the (**a**) body weight, (**b**) body weight gain, (**c**) percent fat mass, (**d**) triglyceride, (**e**) total cholesterol, and (**f**) alanine aminotransferase, ALT of high-fat diet-induced obese mice. Values are presented as mean ± SEM. The significance values [*p* < 0.05] and [*p* < 0.001] are denoted as *, ***, respectively, in comparison with ND. The significance value [*p* < 0.05] is denoted as # in comparison with HFD. Statistical analyses were performed using one-way ANOVA with post-hoc Tukey HSD. ND: normal diet; HFD: high-fat diet; PRO: high-fat diet + *L. rhamnosus* 4B15; PRE: high-fat diet + *C. tricuspidata*; SYN: high-fat diet + *L. rhamnosus* 4B15 + *C. tricuspidata*.

To examine the modulatory effects of *L. rhamnosus* 4B15 and *C. tricuspidata* on body weight and obesity-associated metabolic indices in HFD-induced obese mice, we administered *L. rhamnosus* 4B15, *C. tricuspidata*, and a combination of the two to diet-induced obese mice for 10 weeks (Figure 1). As expected, the HFD significantly increased the body weight and body weight gain (*p* < 0.01; Table S2), as well as total fat mass (*p* < 0.05), and serum levels of TG (*p* < 0.05) and ALT (*p* < 0.05) of the mice compared with the ND mice. Supplementation of *L. rhamnosus* 4B15 and *C. tricuspidata*, or its combination did not significantly affect the body weight and body weight gain of obese mice (*p* > 0.05; Figure 1a,b; Table S2). By contrast, the percent of fat mass was significantly reduced in SYN-fed obese mice (*p* < 0.05; Figure 1c), compared with HFD, PRO, and PRE groups. Similarly, the serum TG level was significantly lowered in the SYN group, in contrast with HFD and other treatments (*p* < 0.05; Figure 1d). Moreover, ALT levels in PRO, PRE, and SYN groups were significantly lower in contrast with the HFD group (*p* < 0.05; Figure 1f). On the other hand, serum T-Chol remained unaffected after administration of *L. rhamnosus* 4B15 and *C. tricuspidata*, or its combination (*p* > 0.05; Figure 1e).

### 3.2. Microbial Richness and Diversity

**Figure 2:**
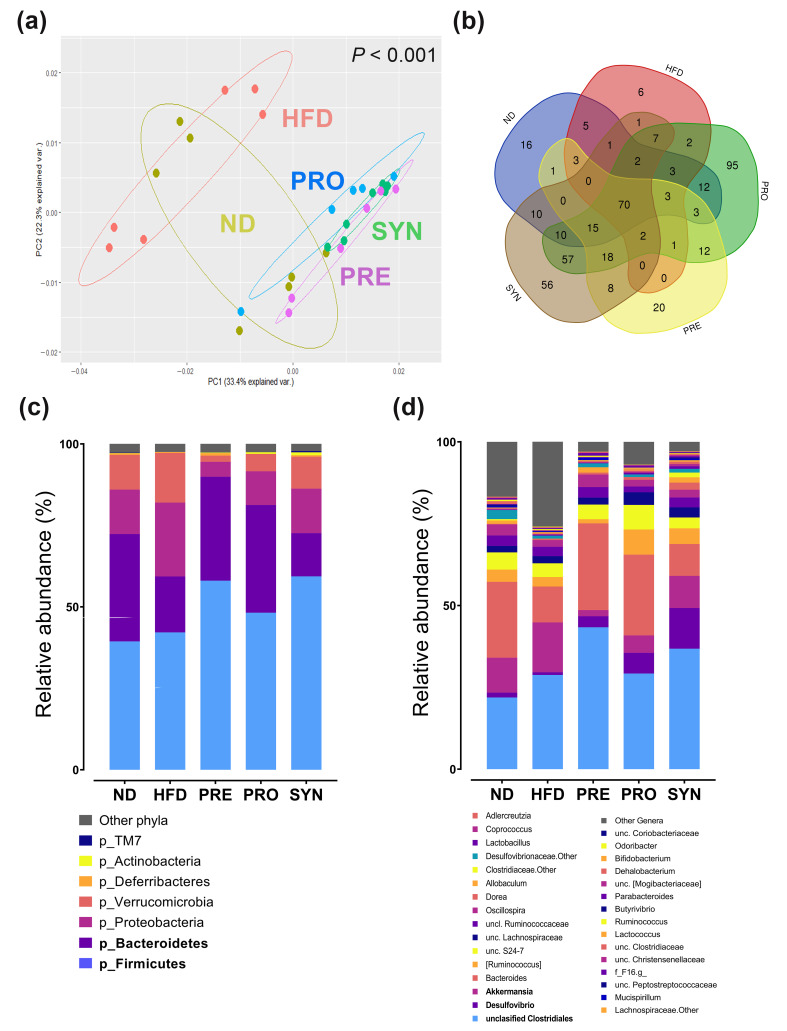
Administration of *L. rhamnosus* 4B15 and *C. tricuspidata* altered the gut microbiota composition. (**a**) Clustering patterns of each group in PCA plot based on Bray–Curtis distance matrix. (**b**) Venn diagram showing the core microbiota of the groups, and unique microbial community of each group. Comparison of gut microbiota composition among all groups at (**c**) phylum and (**d**) genus levels.. Abundance cut-off was set at >0.1%. Abbreviations: ND, normal diet; HFD, high-fat diet; PRO, high-fat diet + *L. rhamnosus* 4B15; PRE, high-fat diet + *C. tricuspidata*; SYN, high-fat diet + *L. rhamnosus* 4B15 + *C. tricuspidata*.

After administering *L. rhamnosus* 4B15 and *C. tricuspidata*, together and individually, we compared the intestinal species richness and diversity among the groups. No significant differences were observed in alpha diversity indices among treatments (ACE, Chao1, Shannon–Weaver, and Simpson) (Table S3). Meanwhile, PCA results revealed that PRO, PRE, and SYN groups clustered discretely from the HFD group (*p* < 0.001; Figure 2a). A pairwise comparison among the groups showed that HFD and SYN were significantly different (*p* < 0.05). Interestingly, these groups overlapped with the ND group, suggesting that they shared features with the ND group.

### 3.3. L. rhamnosus 4B15 and C. tricuspidata Altered the Gut Microbiota of Obese Mice

Consistent with the structural changes in the gut microbiota of the diet-induced obese mice, we also observed changes in the abundance of microbial communities at the phylum and genus levels. We identified 36 bacterial phyla in the ceca (Figure 2c; Table S4). Firmicutes and Bacteroidetes were the most abundant phyla, accounting for ~75% of sequence reads in all groups. The administration of SYN noticeably increased the abundance of Firmicutes (41%) compared with the HFD group. We did not observe any significant difference in the Firmicutes/Bacteroidetes ratio among the groups (Table S4). We found a 63% increase in the abundance of proteobacteria in the HFD group compared with the ND control. By contrast, the abundance of proteobacteria was lower in the PRE, PRO and SYN groups (79%, 54% and 40%, respectively), while actinobacteria expression was drastically elevated in the SYN group (800%; *p* < 0.01) compared with the HFD control.

**Figure 3:**
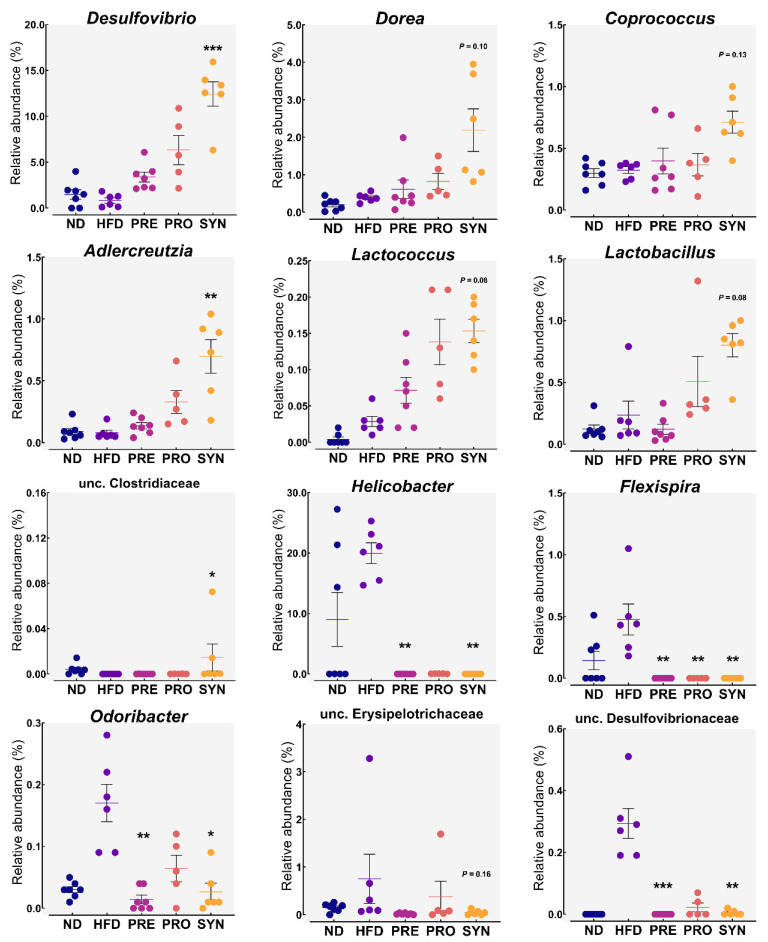
Relative abundances of selected taxa. The significance values [*p* < 0.05], [*p* < 0.01], and [*p* < 0.001] are denoted as *, **, ***, respectively, in comparison with HFD. Values are presented as mean ± SEM. Statistical analyses were performed using Kruskal–Wallis non-parametric test. ND: normal diet; HFD: high-fat diet; PRO: high-fat diet + *L. rhamnosus* 4B15; PRE: high-fat diet + *C. tricuspidata*; SYN: high-fat diet + *L. rhamnosus* 4B15 + *C. tricuspidata*.

At the genus level, we detected 440 bacterial genera in the mouse ceca (Table S5). Of the detected genera, 16, 6, 95, 20, and 56 were exclusive to the ND, HFD, PRO, PRE, and SYN groups, respectively, and 70 core genera were present in all groups (Figure 2b). The most abundant genera in all groups were unclassified Clostridiales, *Bacteroides*, and *Akkermansia* (Figure 2d). The HFD diet enriched several taxa such as *Helicobacter*, unclassified Erysipelotrichaceae and Desulfovibrionaceae, *Flexispira*, and *Odoribacter*. However, supplementation of SYN drastically decreased the abundances of *Helicobacter* (*p* < 0.01), unclassified Desulfovibrionaceae (*p* < 0.05), *Flexispira* (*p* < 0.01), and *Odoribacter* (*p* < 0.05) (Figure 3). In addition, we found that the SYN group mice had a significantly larger population of *Desulfovibrio* (*p* < 0.001), *Adlercreutzia* (*p* < 0.01), and unclassified Clostridiaceae (*p* < 0.05) compared with the HFD group (Figure 3). In addition, the abundances of *Dorea*, *Allobaculum*, *Lactobacillus*, *Coprococcus*, and *Lactococcus* were enriched by SYN supplementation, but not significantly so (Figure 3).

**Figure 4:**
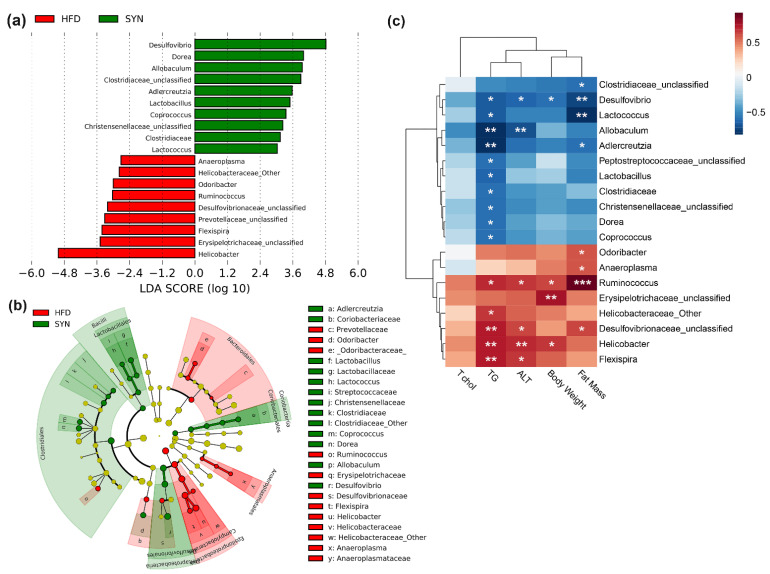
Association between gut microbiota and metabolic parameters. (**a**) Linear discriminant analysis effect size (LEfSe) histogram showing significant differential taxa between HFD (red) and SYN (green) groups, and (**b**) cladogram plot from LEfSe showing the phylogenetic relationships among differential taxa in HFD (red) and SYN (green) groups. (**c**) Heatmap based on Spearman’s correlation between gut microbiota and obesity-associated metabolic parameters. The significance values [*p* < 0.05], [*p* < 0.01], and [*p* < 0.001] is denoted as *, **, and ***, respectively. HFD: high-fat diet; SYN: high-fat diet + *L. rhamnosus* 4B15 + *C. tricuspidata*.

Next, we identified significantly different taxa between HFD and SYN groups using LEfSe [35] (Figure 4a). The LEfSe analysis revealed that *L. rhamnosus* 4B15 and *C. tricuspidata* preferentially increased the abundance of *Desulfovibrio*, *Dorea*, *Allobaculum*, *Adlercreutzia*, *Coprococcus*, *Lactobacillus*, *Lactococcus*, and unclassified Clostridiaceae and Christensenellaceae (*p* < 0.05). By contrast, the HFD elevated the abundance of *Helicobacter*, *Flexispira*, *Anaeroplasma*, *Ruminococcus*, *Odoribacter* and unclassified taxa under family Erysipelotrichaceae, Prevotellaceae, Desulfovibrionaceae, and Helicobacteriaceae (*p* < 0.05). The phylogenetic relationships between these taxa are depicted in a cladogram in Figure 4b.

### 3.4. Synbiotic-Altered Microbiota Is Associated with Metabolic Parameters

Spearman’s correlation coefficients were calculated to determine the associations between the differential taxa and metabolic parameters in the HFD and SYN groups (Figure 4c). We found that *Odoribacter* (*p* < 0.05), *Anaeroplasma* (*p* < 0.05), *Ruminococcus* (*p* < 0.001), and unclassified Desulfovibrionaceae were positively associated with fat mass, while *Ruminococcus* (*p* < 0.05), unclassified Erysipelotrichaceae (*p* < 0.01), and *Helicobacter* (*p* < 0.05) were positively correlated with body weight. Moreover, *Helicobacter* (*p* < 0.01), *Flexispira* (*p* < 0.01, *p* < 0.05, respectively), *Ruminococcus* (*p* < 0.05), and unclassified Desulfovibrionaceae (*p* < 0.01, *p* < 0.05, respectively) were significantly positively correlated with TG and ALT. In contrast, SYN-enriched taxa, such as unclassified Clostridiaceae (*p* < 0.05), *Desulfovibrio* (*p* < 0.01), *Lactococcus* (*p* <0.01), and *Adlercreutzia* (*p* < 0.05) were significantly negatively correlated with adiposity. A significant negative association was observed between *Desulfovibrio* and body weight (*p* < 0.05). In addition, *Desulfovibrio* (*p* < 0.05) and *Allobaculum* (*p* < 0.01) were negatively correlated with ALT. Lastly, TG was negatively correlated with most of SYN-enriched taxa, most notably *Allobaculum* and *Adlercreutzia* (*p* < 0.01). Our results suggest a strong interaction between SYN-modulated taxa and obesity-associated metabolic factors.

### 3.5. Predicted KEGG Functional Pathways in the Gut Microbiome

**Figure 5:**
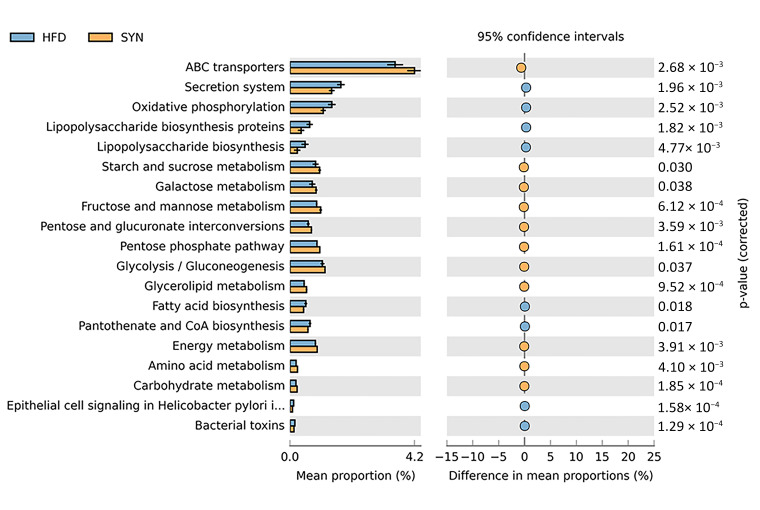
KEGG functional pathways predicted in the gut microbiome of HFD and SYN groups using PICRUSt. Significant differential KEGG pathways between HFD (blue) and SYN (yellow). Statistical analysis was carried out using STAMP software. Abbreviations: HFD, high-fat diet; SYN, high-fat diet + *L. rhamnosus* 4B15 + *C. tricuspidata*.

Functional pathways of the gut microbiome based on KEGG pathways were identified using PICRUSt [36]. A total of 284 KEGG pathways were identified in the gut microbiome of the mice (Table S6), Significantly different pathways between HFD and SYN group are shown in Figure 5. Our results revealed that the administration of *L. rhamnosus* 4B15 and *C. tricuspidata* significantly upregulated several pathways involved in metabolism, including ABC transporters, starch and sucrose metabolism, galactose metabolism, fructose and mannose metabolism, glycerolipid metabolism, glycolysis/gluconeogenesis, energy metabolism, carbohydrate metabolism, and amino acid metabolism (*p* < 0.05). On the other hand, pathways such as the secretion system, oxidative phosphorylation, lipopolysaccharide biosynthesis, fatty acid biosynthesis, epithelial cell signalling in *H. pylori*, and bacterial toxin production were significantly upregulated in HFD mice (*p* < 0.05). This suggests that *L. rhamnosus* 4B15 and *C. tricuspidata* synbiotically influenced the functional pathways expressed in the gut microbiome of obese mice.

## 4. Discussion

Supplementation with *L. rhamnosus* 4B15 [38] or *C. tricuspidata* [29] have been individually shown to have a lowering effect on body weight in previous studies. Although a decreasing trend was observed, neither individual nor combined administration of *L. rhamnosus* 4B15 and *C. tricuspidata* resulted in significant reduction in the body weight of the mouse in the present study. Similarly, previous studies did not observe a significant reduction in body weights of obese mice after supplementation of *C. tricuspidata* [30,39]. Other studies on the effect of probiotic and synbiotic feeding on the body weight were inconclusive as well [40]. Feeding period, concentration, as well as ratio of the synbiotics used may explain the different result observed from the present study [40]. Meanwhile, feeding of HFD increased the fat mass in mice by 2.2-fold in contrast with ND. Excessive fat mass indicates the onset of obesity, and linked to the occurrence of insulin resistance and cardiometabolic diseases [41]. Triglycerides play a major role in the storage and transport of fatty acids, and an abnormal increase in the levels of triglycerides usually leads to cardiovascular disease [42,43,44]. Meanwhile, excessive serum activity of ALT is an indicator of liver damage, including non-alcoholic steatohepatitis [45,46]. In this study, we observed a reduction in fat mass, with serum TG and ALT in PRO, PRE, and SYN treatments. However, significant reduction in TG and fat mass was only observed on SYN-treated mice (*p* < 0.05). Previous data demonstrated the abilities of *L. rhamnosus* 4B15 and *C. tricuspidata* to reduce TG levels and fat mass in animal models [29,30,38]. The results of the present study suggest that the synergistic interaction between *L. rhamnosus* 4B15 and *C. tricuspidata* provides better protection against hypertriglyceridemia and excess adiposity than the probiotic or prebiotic treatment alone. Further investigations are necessary to validate the synbiotic potential of *L. rhamnosus* 4B15 and *C. tricuspidata*.

At phylum level we observed an increase in the abundance of Firmicutes in mice fed with *C. tricuspidata*, alone or in combination with *L. rhamnosus* 4B15. The presence of dietary fibres may explain the increase in abundance of Firmicutes, as several members of this phylum undergo fermentation [47,48]. On the other hand, HFDs can increase the abundance of proteobacteria and decrease the abundance of actinobacteria [49,50]. Many proteobacteria are pathogenic, and can produce endotoxins [51], while actinobacteria play important roles in gut homeostasis [52]. Our results suggest that *L. rhamnosus* 4B15 and *C. tricuspidata* supplementation altered the abundances of these phyla.

In the present study, we found that *Desulfovibrio*, *Dorea*, *Adlercreutzia*, *Allobaculum*, *Coprococcus*, unclassified Clostridiaceae, and *Lactobacillus* were enriched in obese mice fed with *L. rhamnosus* 4B15 and *C. tricuspidata*. Our correlation analysis also revealed that the abundances of these taxa have an inverse correlation with body weight and metabolic parameters. *Desulfovibrio* and *Adlercreutzia* were previously found to be enriched in lean hosts [53,54,55]. *Adlercreutzia* can alleviate diabetes and obesity by producing phenyl-γ-valerolactones; these biomolecules are associated with lean phenotypes [56,57]. *Allobaculum*, *Lactobacillus*, and *Coprococcus* were enriched in hamsters fed with resistant starch [58]. Xiang et al., [59] and Everard et al. [60] also reported that the metabolic syndrome was inversely correlated with the abundance of *Allobaculum*, *Lactobacillus*, and *Coprococcus*; these species produce short-chain fatty acids (SCFAs) [61,62], which are important fermentation by-products due to their roles in body weight regulation, gut permeability, and lipid and glucose metabolism [63,64,65]. In a recent study, Kong et al. [61] found that *Lactococcus* enrichment negatively correlated with obesity. Cross and colleagues [66] found out that *Dorea* negatively correlated with metabolic parameters such as TG, similar with our results.

We also found that *Helicobacter*, *Flexispira*, *Odoribacter*, *Ruminococcus*, unclassified Erysipelotrichaceae, and unclassified Desulfovibrionaceae were enriched in HFD-fed obese mice, and that enrichment of these genera exhibited a strong positive correlation with body weight and metabolic indices. Elevation in the population of *Helicobacter* and *Flexispira* in diet-induced obese animal models have been observed in previous studies [67,68,69]. *Helicobacter* and *Flexispira* are phylogenetically related, and are typically gastrointestinal pathogens [70,71]. In the present study, *Helicobacter* and *Flexispira* were less abundant in the gut of mice treated with *L. rhamnosus* 4B15 and *C. tricuspidata.* This observation is consistent with previous studies on the effect of synbiotics on *Helicobacter* [72]. Several studies have implicated the bacterial family of Erysipelotricaceae with metabolic disorders, such as hypercholesterolemia and gut inflammation [53,73,74]. We also observed an increase in the abundance of *Odoribacter* and *Ruminococcus* in HFD mice, which is in agreement with other studies [75,76]. Our results provide novel insights for the synbiotic potential of *L. rhamnosus* 4B15 and *C. tricuspidata* to modulate the gut bacteria and alleviate obesity-related metabolic syndrome.

Our PICRUSt analysis revealed that several metabolic pathways were significantly upregulated in SYN mice. ABC transporters, the most differential KEGG pathways between SYN and HFD mice, play key roles in the import of nutrients [77] and utilization of prebiotics [78,79]. The elevation of pathways such as galactose metabolism, starch and sucrose metabolism, fructose and mannose metabolism, and glycolysis/gluconeogenesis, as well as energy, carbohydrate, and amino acid metabolism, suggests that *L. rhamnosus* 4B15 and *C. tricuspidata* increased energy utilization, which in turn counteracted the obesogenic effects of the HFD [80,81,82]. Increased sugar and amino acid metabolism increases SCFA production, which in turn leads to a normal lipid profile and less inflammation in the gut [83,84]. SCFAs also play roles in glucose homeostasis and appetite regulation [65]. Glycerolipid metabolism has a vital role in the regulation of lipolysis and glucose homeostasis; dysregulation of the glycerolipid pathway can lead to obesity and type 2 diabetes [85]. This suggests that the administration of *L. rhamnosus* 4B15 and *C. tricuspidata* enriched metabolic pathways that can counteract the effects of HFDs.

We also observed the upregulation of several KEGG pathways in mice fed HFDs. Notably, lipopolysaccharide biosynthesis, epithelial cell signalling in *H. pylori* and bacterial toxin pathways were upregulated. In previous studies, the lipopolysaccharide biosynthesis pathway was enriched in patients with *H. pylori* infections [86], while the intensity of epithelial cell signalling in *H. pylori* pathway coincided with an increase in *Helicobacter* abundance [87]. These results reflect our observations of increase of *Helicobacter*, and its close relative *Flexispira*, as well as the upregulation of lipopolysaccharide biosynthesis and epithelial cell signalling pathways in the HFD group. We hypothesise that the high abundance of *Helicobacter* and *Flexispira* triggered the upregulation of the bacterial toxins pathway in the gut microbiota. Expectedly, there was an upregulation of fatty acid biosynthesis in HFD-fed mice. Excessive lipogenesis contributes to the pathogenesis of obesity and other related metabolic diseases. Thus, inhibition of integral enzymes, such as fatty acid synthase, is a potential therapeutic target for the treatment of obesity [88].

## 5. Conclusions

In conclusion, the results of the current study demonstrated that the combination of *L. rhamnosus* 4B15 and *C. tricuspidata* exerted a synbiotic effect on the gut microbiota and metabolic health of the obese mice. Administration of *L. rhamnosus* 4B15 and *C. tricuspidata* alleviated excess adiposity, and increased serum TG and ALT levels induced by HFD. The synbiotic treatment also modulated the gut microbiota, notably *Desulfovibrio*, *Dorea*, *Adlercreutzia*, *Allobaculum*, *Coprococcus*, unclassified Clostridiaceae, *Lactobacillus*, *Helicobacter*, *Flexispira*, *Odoribacter*, *Ruminococcus*, unclassified Erysipelotrichaceae, and unclassified Desulfovibrionaceae. These taxa revealed a strong correlation with body weight and metabolic parameters. Lastly, *L. rhamnosus* 4B15 and *C. tricuspidata* upregulated metabolic pathways known to improve metabolic health. Further investigations are needed to understand the mechanisms driving the synbiotic effect of *C. tricuspidata* and *L. rhamnosus* 4B15.

## Data Availability

All standard sequence format (.fastq) files generated by Illumina Miseq containing all raw sequence reads have been deposited at the National Center for Biotechnology Information (NCBI) Sequence Read Archive (SRA) (BioProject accession number: PRJNA691724).

## Supplementary Materials

The following are available online at https://www.mdpi.com/article/10.3390/microorganisms9091908/s1, Figure S1: Schematic diagram showing the schedule for the feeding experiment. Table S1: Composition of experimental mouse diets. Table S2: Weekly measurements of body weight and feed intake of experimental C57BL/N6 mice. Table S3: Comparison of alpha-diversity indices of the gut microbiota of C57BL/N6 mice. Table S4: Relative abundance of taxonomic groups at phylum level (cut-off > 0.1%). Table S5: Relative abundance of taxonomic groups at genus level (cut-off > 0.1%). Table S6: Relative abundance of predicted KEGG pathways in mice gut microbiota (cut-off > 0.1%).

## Abbreviations

ACE	Abundance-based Estimator
HFD	High fat diet
KEGG	Kyoto Encyclopedia of Genes and Genomes
LEfSe	Linear Discriminant Analysis Effect Size
PCA	Principal Component Analysis
PICRUSt	Phylogenetic Investigation of Communities by Reconstruction of Unobserved States
QIIME	Quantitative Insights Into Microbial Ecology
