# Supplementary material for: Cudrania tricuspidata Combined with Lacticaseibacillus rhamnosus Modulate Gut Microbiota and Alleviate Obesity-Associated Metabolic Parameters in Obese Mice
Source: Microorganisms. 2021 Sep 8;9(9):1908. doi: 10.3390/microorganisms9091908 (PMC8468176; doi:10.3390/microorganisms9091908)
Supplement: Supplementary file 1 [file microorganisms-09-01908-s001.zip › microorganisms-1310292-supplementary.pdf]

***Cudrania tricuspidata* Combined with *Lacticaseibacillus rhamnosus* Modulate Gut Microbiota and Alleviate Obesity-associated Metabolic Parameters in Obese Mice**

Ju Kyoung Oh, Robie Vasquez, In-Chan Hwang, Ye Na Oh, Sang Hoon Kim, Shin Ho Kang, Jae Yeon Joung, Nam Su Oh, Sejeong Kim, Yohan Yoon, and Dae-Kyung Kang

Supplementary Data

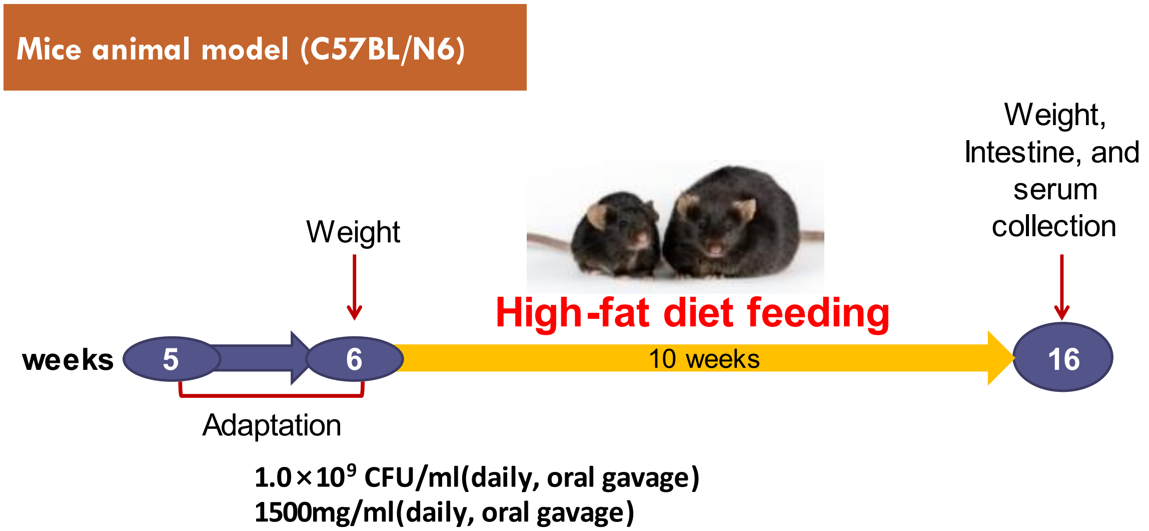

Group	Diet	Treatment
Normal group(NORM, n=7)	10% fat	PBS
Control group(HFD, n=6)	45% fat	PBS
Prebiotic group(PRE, n=7)	45% fat	*Cudrania tricuspidata*
Probiotic group(PRO, n=5)	45% fat	*Lacticaseibacillus rhamnosus* 4B15
Synbiotic group (SYN, n=6)	45% fat	*C. tricuspidata* + *L. rhamnosus* 4B15

Figure S1. Schematic diagram showing the schedule for the feeding experiment. After a 1-week acclimation period, 31 six-week-old male C57BL/N6 mice were divided into five diet groups: normal diet (ND) group; high-fat diet (HFD) group; probiotic (PRO) group; prebiotic (PRE) group; and synbiotic (SYN) group. After 10 weeks of feeding, intestinal contents were collected and subjected to further analyses.

Table S1. Composition of experimental mouse diets.

Item	ND	HFD
Ingredients (g/kg)		
Casein	210.0	245.0
L-Cystine	3.0	3.5
Maltodextrin	50.0	115.0
Sucrose	325.0	200.0
Lard	20.0	195.0
Soybean oil	20.0	30.0
Cellulose	37.15	58.0
Corn starch	280.0	85.0
Mineral Mix[1]	35.0	43.0
$CaH(PO_4)_2$	2.0	3.4
Vitamin Mix[2]	15.0	19.0
Choline bitartrate	2.75	3.0
Food color	0.1	0.1
Chemical composition: g (w/w) (kcal)		
Protein	18.8(20.1)	21.7(19.0)
Carbohydrate	64.7(69.8)	41.4(36.2)
Fat	4.2(10.2)	22.7(44.8)
kcal/g	3.6	4.6

ND, Normal diet (TD.94048 AIN-93M Purified Diet.); HFD, High-fat diet (TD.06415 Adjusted Calories Diet (45 kcal/fat))

[1]Mineral mix = AIN (American Institute of Nutrition)-93G mineral mixture

[2]Vitamin mix = AIN-93G vitamin mixture.

Table S2. Weekly measurements of body weight and feed intake of experimental C57BL/N6 mice.

Groups	Items	Weeks										
		0	1	2	3	4	5	6	7	8	9	10
ND	Weight, g	20.48±0.29	21.06±0.37	22.13±0.22	22.99±0.19	23.89±0.78	25.31±0.69	25.89±0.75	26.44±0.77	26.58±0.70	27.51±0.77	25.84±0.69
	Feed intake, g		75.64	75.80	69.00	71.20	80.61	79.16	77.89	33.04	40.13	34.01
HFD	Weight, g	20.81±0.32	21.57±0.28	22.53±0.49	23.87±0.40	25.31±0.49	26.33±0.68	28.14±0.72	29.46±0.75	31.23±0.87	31.73±0.86	32.14±0.92
	Feed intake, g		47.50	53.50	57.61	57.65	57.64	63.92	61.28	62.06	56.99	62.51
PRE	Weight, g	20.95±0.35	22.07±0.44	23.31±0.58	24.86±0.70	26.12±0.74	26.55±0.79	27.89±0.95	29.08±0.99	29.58±0.84	29.81±0.74	30.75±0.79
	Feed intake, g		56.69	67.83	72.15	72.38	68.56	74.95	73.01	73.34	60.18	64.56
PRO	Weight, g	20.43±0.43	21.71±0.46	22.42±0.45	22.29±0.48	24.19±0.38	25.45±0.28	26.50±0.58	27.82±0.65	29.06±0.57	30.57±0.48	31.69±0.46
	Feed intake, g		72,16	65.14	65.57	81.77	76.62	70.15	74.17	70.21	69.32	68.60
SYN	Weight, g	20.50±0.52	22.28±0.59	22.74±0.38	23.95±0.57	25.16±0.79	25.92±0.79	27.01±1.46	27.69±1.08	28.66±1.15	29.96±1.30	30.37±1.48
	Feed intake, g		68.47	66.91	65.34	69.73	70.00	74.30	73.59	79.13	71.38	73.57

Body weight values were expressed as mean ± Standard Error of Mean (SEM).
Abbreviations: ND, normal diet; HFD, high-fat diet; PRO, high-fat diet + *L. rhamnosus* 4B15; PRE, high-fat diet + *C. tricuspidata*; SYN, high-fat diet + *L. rhamnosus* 4B15 + *C. tricuspidata*.

Table S3. Comparison of alpha-diversity indices of the gut microbiota of C57BL/N6 mice.

Items	Groups[1]					*P*-value[2]
	ND	HFD	PRE	PRO	SYN	
ACE	5261±969	5209±427	5933±584	4410±302	3989±559	0.25
Chao1	4810±899	4652±403	5453±538	4032±255	3633±502	0.13
Shannon-Weaver	6.08±0.24	5.77±0.18	6.10±0.32	5.82±0.32	6.62±0.34	0.30
Simpson	0.91±0.01	0.900±0.01	0.91±0.01	0.90±0.02	0.94±0.01	0.10

[1]Values were expressed as mean ± Standard Error of Mean.
[2]*P*-values for were calculated using one-way ANOVA.
Abbreviations: ND, normal diet; HFD, high-fat diet; PRO, high-fat diet + *L. rhamnosus* 4B15; PRE, high-fat diet + *C. tricuspidata*; SYN, high-fat diet + *L. rhamnosus* 4B15 + *C. tricuspidata.*

Table S4. Relative abundance of taxonomic groups at phylum level (cut-off > 0.1).

Phyla	Relative Abundance, %				
	ND	HFD	PRE	PRO	SYN
Firmicutes	39.40	42.15	58.04	48.21	59.37
Bacteroidetes	32.94	17.17	31.86	33.03	13.24
F/B ratio	2.42	2.72	1.92	1.81	4.40
Proteobacteria	13.63	22.66	4.61	10.35	13.67
Verrucomicrobia	10.66	15.25	1.88	5.34	9.83
Deferribacteres	0.44	0.29	0.79	0.01	0.42
Actinobacteria	0.11	0.10	0.19	0.51	0.88
TM7	0.14	0.15	0.04	0.004	0.39
Other Phyla	2.69	2.23	2.59	2.56	2.20

Values were expressed as mean.
Abbreviations: ND, normal diet; HFD, high-fat diet; PRO, high-fat diet + *L. rhamnosus* 4B15; PRE, high-fat diet + *C. tricuspidata*; SYN, high-fat diet + *L. rhamnosus* 4B15 + *C. tricuspidata*

Table S5. Relative abundance of taxonomic groups at genus level (cut-off > 0.1).

Genus	Relative Abundance, %				
	ND	HFD	PRE	PRO	SYN
unclassified Clostridiales	21.91	28.76	43.34	29.23	36.80
Bacteroides	23.20	10.97	26.49	24.68	9.73
Akkermansia	10.66	15.25	1.88	5.33	9.82
Helicobacter	8.99	19.97	0.00	0.04	0.00
unclassified S24-7	5.27	4.18	4.48	7.50	3.31
Desulfovibrio	1.47	0.83	3.38	6.32	12.43
[Ruminococcus]	3.73	2.89	1.27	7.66	4.78
unclassified Ruminococcaceae	3.19	2.86	3.25	1.77	3.02
Oscillospira	3.38	2.06	3.82	2.06	2.35
unclassified Lachnospiraceae	1.95	2.19	2.09	3.86	3.11
unclassified Desulfovibrionaceae (other)	2.79	0.92	1.16	0.79	1.11
[Prevotella]	3.73	0.59	0.00	0.02	0.01
Allobaculum	1.00	0.03	1.63	0.00	1.61
Dorea	0.20	0.39	0.61	0.82	2.19
unclassified Helicobacteraceae	0.00	0.01	0.00	2.96	0.00
Parabacteroides	0.50	0.19	0.75	0.62	0.13
Mucispirillum	0.44	0.29	0.79	0.01	0.42
Coprococcus	0.30	0.33	0.40	0.36	0.71
unclassified Clostridiaceae (other)	0.41	0.00	0.00	0.01	1.46
unclassified Lachnospiraceae	0.25	0.43	0.35	0.39	0.46
Lactobacillus	0.12	0.24	0.12	0.51	0.80
Ruminococcus	0.45	0.34	0.34	0.16	0.15
unclassified Clostridiaceae	0.84	0.07	0.04	0.11	0.29
Adlercreutzia	0.09	0.08	0.14	0.33	0.70
unclassified Erysipelotrichaceae	0.15	0.75	0.02	0.38	0.04
unclassified Christensenellaceae	0.08	0.08	0.08	0.06	0.34
Flexispira	0.14	0.48	0.00	0.00	0.00
Lactococcus	0.00	0.03	0.07	0.14	0.15
unclassified Desulfovibrionaceae	0.00	0.29	0.00	0.02	0.01
Odoribacter	0.03	0.17	0.02	0.06	0.03
Bifidobacterium	0.00	0.01	0.04	0.02	0.09
Other Genera	4.70	4.33	3.44	3.80	3.96

Values were expressed as mean.

Abbreviations: ND, normal diet; HFD, high-fat diet; PRO, high-fat diet + *L. rhamnosus* 4B15; PRE, high-fat diet + *C. tricuspidata*; SYN, high-fat diet + *L. rhamnosus* 4B15 + *C. tricuspidata*

Table S6. Relative abundance of predicted KEGG pathways in mice gut microbiota (cut-off > 0.1).

KEGG Pathways			Relative Abundance, %	
Level 1	Level 2	Level 3	HFD	SYN
Environmental Information Processing	Membrane Transport	Transporters	7.02	8.32
Environmental Information Processing	Membrane Transport	ABC transporters	3.22	3.81
Genetic Information Processing	Replication and Repair	DNA repair and recombination proteins	2.50	2.55
Metabolism	Nucleotide Metabolism	Purine metabolism	2.06	2.05
Environmental Information Processing	Signal Transduction	Two-component system	2.04	2.03
Genetic Information Processing	Translation	Ribosome	2.08	1.95
Genetic Information Processing	Transcription	Transcription factors	1.55	1.89
Metabolism	Enzyme Families	Peptidases	1.72	1.77
Metabolism	Nucleotide Metabolism	Pyrimidine metabolism	1.61	1.65
Cellular Processes	Cell Motility	Bacterial motility proteins	1.76	1.51
Metabolism	Carbohydrate Metabolism	Amino sugar and nucleotide sugar metabolism	1.39	1.44
Genetic Information Processing	Replication and Repair	Chromosome	1.35	1.37
Metabolism	Amino Acid Metabolism	Amino acid related enzymes	1.43	1.36
Metabolism	Energy Metabolism	Methane metabolism	1.21	1.30
Environmental Information Processing	Membrane Transport	Secretion system	1.56	1.28
Metabolism	Amino Acid Metabolism	Arginine and proline metabolism	1.34	1.25
Other	Cellular Processes and Signaling	Other ion-coupled transporters	1.18	1.21
Genetic Information Processing	Translation	Ribosome Biogenesis	1.18	1.19
Genetic Information Processing	Replication and Repair	DNA replication proteins	1.05	1.09
Genetic Information Processing	Translation	Aminoacyl-tRNA biosynthesis	1.17	1.08
Metabolism	Carbohydrate Metabolism	Glycolysis / Gluconeogenesis	0.99	1.07
Metabolism	Metabolism of Cofactors and Vitamins	Porphyrin and chlorophyll metabolism	0.85	1.04
Metabolism	Energy Metabolism	Oxidative phosphorylation	1.28	1.02
Metabolism	Carbohydrate Metabolism	Pyruvate metabolism	1.04	0.99
Metabolism	Amino Acid Metabolism	Alanine, aspartate and glutamate metabolism	0.99	0.99

Genetic Information Processing	Transcription	Transcription machinery	0.99	0.96
Metabolism	Carbohydrate Metabolism	Fructose and mannose metabolism	0.82	0.94
Metabolism	Carbohydrate Metabolism	Pentose phosphate pathway	0.83	0.92
Metabolism	Carbohydrate Metabolism	Starch and sucrose metabolism	0.79	0.91
Genetic Information Processing	Folding, Sorting and Degradation	Chaperones and folding catalysts	0.99	0.90
Metabolism	Energy Metabolism	Carbon fixation pathways in prokaryotes	1.02	0.87
Metabolism	Amino Acid Metabolism	Cysteine and methionine metabolism	0.82	0.85
Other	Metabolism	Energy metabolism	0.78	0.84
Other	Genetic Information Processing	Translation proteins	0.84	0.84
Metabolism	Amino Acid Metabolism	Glycine, serine and threonine metabolism	0.79	0.81
Metabolism	Carbohydrate Metabolism	Galactose metabolism	0.68	0.81
Other	Genetic Information Processing	Protein folding and associated processing	0.89	0.79
Genetic Information Processing	Replication and Repair	Homologous recombination	0.78	0.78
Metabolism	Amino Acid Metabolism	Lysine biosynthesis	0.77	0.77
Metabolism	Amino Acid Metabolism	Phenylalanine, tyrosine and tryptophan biosynthesis	0.82	0.77
Other	Genetic Information Processing	Replication, recombination and repair proteins	0.75	0.76
Cellular Processes	Cell Motility	Bacterial chemotaxis	0.77	0.74
Other	Cellular Processes and Signaling	Sporulation	0.51	0.74
Genetic Information Processing	Replication and Repair	Mismatch repair	0.73	0.73
Cellular Processes	Cell Motility	Flagellar assembly	0.92	0.73
Metabolism	Amino Acid Metabolism	Valine, leucine and isoleucine biosynthesis	0.79	0.72
Metabolism	Glycan Biosynthesis and Metabolism	Peptidoglycan biosynthesis	0.69	0.71
Metabolism	Energy Metabolism	Nitrogen metabolism	0.65	0.69
Metabolism	Carbohydrate Metabolism	Pentose and glucuronate interconversions	0.56	0.66
Metabolism	Carbohydrate Metabolism	Butanoate metabolism	0.60	0.61
Metabolism	Energy Metabolism	Carbon fixation in photosynthetic organisms	0.60	0.60
Environmental Information Processing	Membrane Transport	Bacterial secretion system	0.74	0.59

Genetic Information Processing	Replication and Repair	DNA replication	0.59	0.59
Metabolism	Metabolism of Cofactors and Vitamins	One carbon pool by folate	0.56	0.57
Metabolism	Carbohydrate Metabolism	Glyoxylate and dicarboxylate metabolism	0.58	0.56
Metabolism	Metabolism of Cofactors and Vitamins	Pantothenate and CoA biosynthesis	0.61	0.55
Metabolism	Lipid Metabolism	Lipid biosynthesis proteins	0.58	0.54
Metabolism	Lipid Metabolism	Glycerophospholipid metabolism	0.59	0.53
Metabolism	Amino Acid Metabolism	Histidine metabolism	0.57	0.53
Metabolism	Carbohydrate Metabolism	Citrate cycle (TCA cycle)	0.66	0.53
Genetic Information Processing	Folding, Sorting and Degradation	Protein export	0.57	0.52
Metabolism	Lipid Metabolism	Glycerolipid metabolism	0.43	0.51
Metabolism	Carbohydrate Metabolism	Propanoate metabolism	0.54	0.47
Cellular Processes	Cell Growth and Death	Cell cycle - Caulobacter	0.46	0.47
Metabolism	Metabolism of Terpenoids and Polyketides	Terpenoid backbone biosynthesis	0.48	0.46
Other	Cellular Processes and Signaling	Membrane and intracellular structural molecules	0.61	0.46
Genetic Information Processing	Translation	Translation factors	0.48	0.46
Metabolism	Metabolism of Cofactors and Vitamins	Thiamine metabolism	0.42	0.45
Genetic Information Processing	Folding, Sorting and Degradation	RNA degradation	0.47	0.44
Other	Cellular Processes and Signaling	Signal transduction mechanisms	0.42	0.43
Metabolism	Glycan Biosynthesis and Metabolism	Other glycan degradation	0.42	0.43
Metabolism	Lipid Metabolism	Fatty acid biosynthesis	0.49	0.42
Genetic Information Processing	Replication and Repair	Base excision repair	0.42	0.41
Environmental Information Processing	Membrane Transport	Phosphotransferase system (PTS)	0.21	0.41
Metabolism	Enzyme Families	Protein kinases	0.37	0.38
Genetic Information Processing	Replication and Repair	Nucleotide excision repair	0.39	0.37
Metabolism	Metabolism of Other Amino Acids	Selenocompound metabolism	0.36	0.35
Metabolism	Glycan Biosynthesis and Metabolism	Lipopolysaccharide biosynthesis proteins	0.60	0.34
Metabolism	Metabolism of Cofactors and Vitamins	Nicotinate and nicotinamide metabolism	0.30	0.34

Metabolism	Energy Metabolism	Photosynthesis proteins	0.36	0.33
Cellular Processes	Cell Motility	Cytoskeleton proteins	0.31	0.33
Metabolism	Lipid Metabolism	Sphingolipid metabolism	0.31	0.33
Metabolism	Biosynthesis of Other Secondary Metabolites	Streptomycin biosynthesis	0.32	0.32
Metabolism	Energy Metabolism	Photosynthesis	0.35	0.32
Metabolism	Metabolism of Cofactors and Vitamins	Folate biosynthesis	0.42	0.31
Metabolism	Carbohydrate Metabolism	C5-Branched dibasic acid metabolism	0.32	0.31
Metabolism	Amino Acid Metabolism	Tyrosine metabolism	0.28	0.30
Metabolism	Xenobiotics Biodegradation and Metabolism	Drug metabolism - other enzymes	0.27	0.30
Metabolism	Glycan Biosynthesis and Metabolism	Glycosyltransferases	0.34	0.30
Other	Cellular Processes and Signaling	Inorganic ion transport and metabolism	0.33	0.28
Metabolism	Energy Metabolism	Sulfur metabolism	0.32	0.27
Other	Cellular Processes and Signaling	Pores ion channels	0.36	0.27
Metabolism	Metabolism of Other Amino Acids	Cyanoamino acid metabolism	0.26	0.27
Metabolism	Metabolism of Terpenoids and Polyketides	Prenyltransferases	0.29	0.26
Genetic Information Processing	Folding, Sorting and Degradation	Sulfur relay system	0.30	0.25
Other	Metabolism	Amino acid metabolism	0.19	0.23
Metabolism	Amino Acid Metabolism	Valine, leucine and isoleucine degradation	0.20	0.23
Other	Metabolism	Carbohydrate metabolism	0.18	0.22
Metabolism	Glycan Biosynthesis and Metabolism	Lipopolysaccharide biosynthesis	0.46	0.22
Other	Cellular Processes and Signaling	Cell motility and secretion	0.31	0.22
Metabolism	Lipid Metabolism	Fatty acid metabolism	0.19	0.21
Metabolism	Amino Acid Metabolism	Phenylalanine metabolism	0.19	0.21
Cellular Processes	Transport and Catabolism	Lysosome	0.23	0.20
Metabolism	Xenobiotics Biodegradation and Metabolism	Chloroalkane and chloroalkene degradation	0.17	0.20

Metabolism	Metabolism of Terpenoids and Polyketides	Polyketide sugar unit biosynthesis	0.19	0.20
Metabolism	Metabolism of Cofactors and Vitamins	Ubiquinone and other terpenoid-quinone biosynthesis	0.24	0.19
Metabolism	Xenobiotics Biodegradation and Metabolism	Benzoate degradation	0.18	0.19
Organismal Systems	Environmental Adaptation	Plant-pathogen interaction	0.18	0.17
Metabolism	Carbohydrate Metabolism	Ascorbate and aldarate metabolism	0.14	0.17
Metabolism	Metabolism of Other Amino Acids	Glutathione metabolism	0.22	0.17
Metabolism	Metabolism of Cofactors and Vitamins	Riboflavin metabolism	0.24	0.17
Metabolism	Metabolism of Cofactors and Vitamins	Vitamin B6 metabolism	0.17	0.17
Cellular Processes	Transport and Catabolism	Peroxisome	0.16	0.17
Metabolism	Xenobiotics Biodegradation and Metabolism	Naphthalene degradation	0.14	0.16
Metabolism	Amino Acid Metabolism	Tryptophan metabolism	0.18	0.16
Genetic Information Processing	Translation	RNA transport	0.11	0.15
Metabolism	Carbohydrate Metabolism	Inositol phosphate metabolism	0.15	0.15
Metabolism	Biosynthesis of Other Secondary Metabolites	Phenylpropanoid biosynthesis	0.13	0.15
Metabolism	Glycan Biosynthesis and Metabolism	Glycosphingolipid biosynthesis - globo series	0.15	0.14
Genetic Information Processing	Transcription	RNA polymerase	0.14	0.14
Metabolism	Lipid Metabolism	Biosynthesis of unsaturated fatty acids	0.19	0.14
Metabolism	Metabolism of Other Amino Acids	beta-Alanine metabolism	0.16	0.14
Human Diseases	Infectious Diseases	Tuberculosis	0.14	0.14
Metabolism	Xenobiotics Biodegradation and Metabolism	Aminobenzoate degradation	0.15	0.14
Other	Metabolism	Lipid metabolism	0.14	0.13
Metabolism	Metabolism of Cofactors and Vitamins	Biotin metabolism	0.18	0.13
Environmental Information Processing	Signaling Molecules and Interaction	Bacterial toxins	0.15	0.13
Metabolism	Metabolism of Terpenoids and Polyketides	Biosynthesis of ansamycins	0.11	0.13

Metabolism	Amino Acid Metabolism	Lysine degradation	0.15	0.12
Metabolism	Biosynthesis of Other Secondary Metabolites	Novobiocin biosynthesis	0.12	0.12
Metabolism	Metabolism of Other Amino Acids	D-Glutamine and D-glutamate metabolism	0.13	0.12
Metabolism	Metabolism of Other Amino Acids	Taurine and hypotaurine metabolism	0.14	0.12
Other	Metabolism	Metabolism of cofactors and vitamins	0.13	0.11
Metabolism	Metabolism of Terpenoids and Polyketides	Tetracycline biosynthesis	0.14	0.10
Metabolism	Metabolism of Terpenoids and Polyketides	Limonene and pinene degradation	0.11	0.10
Metabolism	Metabolism of Other Amino Acids	D-Alanine metabolism	0.11	0.10
Metabolism	Biosynthesis of Other Secondary Metabolites	Tropane, piperidine and pyridine alkaloid biosynthesis	0.10	0.10
Organismal Systems	Nervous System	Glutamatergic synapse	0.09	0.10
Metabolism	Glycan Biosynthesis and Metabolism	Glycosaminoglycan degradation	0.13	0.10
Other	Genetic Information Processing	Restriction enzyme	0.15	0.09
Environmental Information Processing	Signal Transduction	Phosphatidylinositol signaling system	0.11	0.09
Human Diseases	Infectious Diseases	Epithelial cell signaling in Helicobacter pylori infection	0.11	0.08
Other	Other	Other	9.40	9.35

Abbreviations: KEGG, Kyoto Encyclopedia of Genes and Genomes; HFD, high-fat diet; SYN, high-fat diet + *L. rhamnosus* 4B15 + *C. tricuspidata.*
